# Systematic Review: Representativeness of Participants in RCTs of Acetylcholinesterase Inhibitors

**DOI:** 10.1371/journal.pone.0124500

**Published:** 2015-05-01

**Authors:** Anne Leinonen, Marjaana Koponen, Sirpa Hartikainen

**Affiliations:** University of Eastern Finland, Faculty of Health Sciences, School of Pharmacy, Kuopio, Finland; INRCA, ITALY

## Abstract

**Objective:**

To determine whether there are differences in age and sex distribution and presence of comorbidities between participants included in randomized controlled trials of acetylcholinesterase inhibitors and nationwide cohort of persons with Alzheimer’s disease.

**Methods:**

PubMed, Scopus and Cochrane Library databases were searched for original articles from their inception to January 4, 2015. Double-blind randomized controlled trials with donepezil, rivastigmine or galantamine compared to placebo in participants with Alzheimer’s disease were included. Data from a nationwide cohort of persons with clinically verified diagnoses of Alzheimer’s disease was defined as a reference population.

**Results:**

128 full-text articles were assessed for eligibility, 31 of them fulfilled criteria. Mean age of participants in randomized controlled trials (n = 15,032) was 5.8 years lower (95% CI 5.7 to 5.9, P < 0.001), compared to the mean age of 79.7 years in the reference population with Alzheimer’s disease (n = 28,093). Most of the articles did not report age distribution of participants. The proportion of women was 63.2% (9,475/14,991) in randomized controlled trials and 67.8% (19,043/28,093) (P < 0.001) in the reference population. Information on comorbidities and use of concomitant drugs were lacking or poorly reported in most articles.

**Conclusions:**

There is a discrepancy between participants in randomized controlled trials of acetylcholinesterase inhibitors and real-life population with Alzheimer’s disease. Participants in randomized controlled trials were significantly younger. Further, more detailed reporting of age distribution, comorbidities and concomitant drugs would be important information for clinicians when evaluating conclusions from randomized controlled trials to real-life practice. The existing recommendations of inclusion of older people should be followed to ensure safe pharmacotherapy for older people.

## Introduction

Older people are the fastest growing group in the population [1]. However, older people are underrepresented in clinical trials, especially those aged over 75 years, with multiple comorbid conditions, concomitant drugs and/or frailty [2]. Underrepresentation of older people has been identified in several therapeutic areas such as arthritis [3], oncology [4,5], cardiovascular diseases [2,6,7], and depression [8]. Older people fulfill inclusion criteria of clinical trials less likely than younger ones [9,10]. In geriatric population, the proportion of women is higher than men, therefore the majority of subjects enrolled into the clinical trials should to be women [11].

Food and Drug Administration (FDA) and European Medicines Agency (EMA) have had concerns about the generalizability of clinical trial results into older age groups, since the late 1980s [12]. Thus, the International Conference on Harmonization guideline in geriatric patients (ICH E7) was adopted in 1994 in the US, Europe and Japan and it recommended that participants in clinical trials should represent the target population for the drug [13]. Accordingly in Europe, EMA launched the geriatric medicine strategy in 2011 [2,14].

About 35.6 million people were living with dementia in 2010 and the number is projected to nearly double by 2030 and to be over 115 million in 2050 due to global population aging [15]. The aim of this systematic review was to study the representativeness of participants in published RCTs of acetylcholinesterase inhibitors. RCT participants were compared to a real-life user population with Alzheimer’s disease (AD). Similarity was assessed in relation to age, sex and presence of comorbidities and concomitant drugs.

## Materials and Methods

### Literature search strategy

The literature search focused on identifying articles concerning double-blind, placebo-controlled, randomized trials of donepezil, rivastigmine and galantamine that were published before September 3, 2013 without language restriction. The search was updated on January 4, 2015. One author (AL) performed an electronic literature search from PubMed, Scopus and Cochrane Library databases with the help of information specialist (HL) from the University Library of Eastern Finland. The following Medical Subject Heading terms and keywords were used: alzheimer disease, alzheimer*, randomized controlled trial, random allocation, randomly allocated, randomi*, double-blind method, double blind, randomi* controlled trial*, random* W/3 allocat*, donepezil, aricept, E2020, E-2020, rivastigmine, exelon, ENA, SDZ ENA 713, galantamin*, galanthamin*, reminyl, placebo* (S1 Appendix).

This systematic review follows the recommendations of the PRISMA statement [16] (see S1 Checklist). Article selection criteria are defined in Table 1. According to these criteria, we included studies with any dose, dosage form and treatment duration or with any severity stage of Alzheimer’s disease enrolling at least 40 participants. Imaging studies that fulfilled our selection criteria were also included. We only included articles presenting original data. In case of several articles from the same study population, the first article with original data was selected.

**Table 1:** Inclusion and exclusion criteria for article selection.

**Inclusion criteria**
I. We included published, double-blind, placebo-controlled, randomized controlled trials (RCTs) involving participants with a diagnosis of probable Alzheimer’s disease (AD).
II. The diagnosis of probable AD was made according to the National Institute of Neurological and Communicative Disorders and Stroke and the Alzheimer’s Disease and Related Disorders Association (NINCDS-ADRDA) criteria or the Diagnostic and Statistical Manual of Mental Disorders (DSM)-IV criteria.
III. Participants were treated with one of the acetylcholinesterase inhibitors (AChEIs): donepezil, rivastigmine or galantamine, compared with placebo.
IV. Participants were community-dwelling or proportion of participants living in a residential care facility was no more than 2.5%.
V. All articles presenting original data were included.
**Exclusion criteria**
I. Studies including participants with several dementing disorders if demographics and results were not reported separately for participants with AD.
II. Studies including fewer than 40 participants.
III. Studies where participants received open label AChEI before randomization were excluded.
IV. Comparison studies of AChEIs were excluded.

### Data extraction

**Fig 1:**
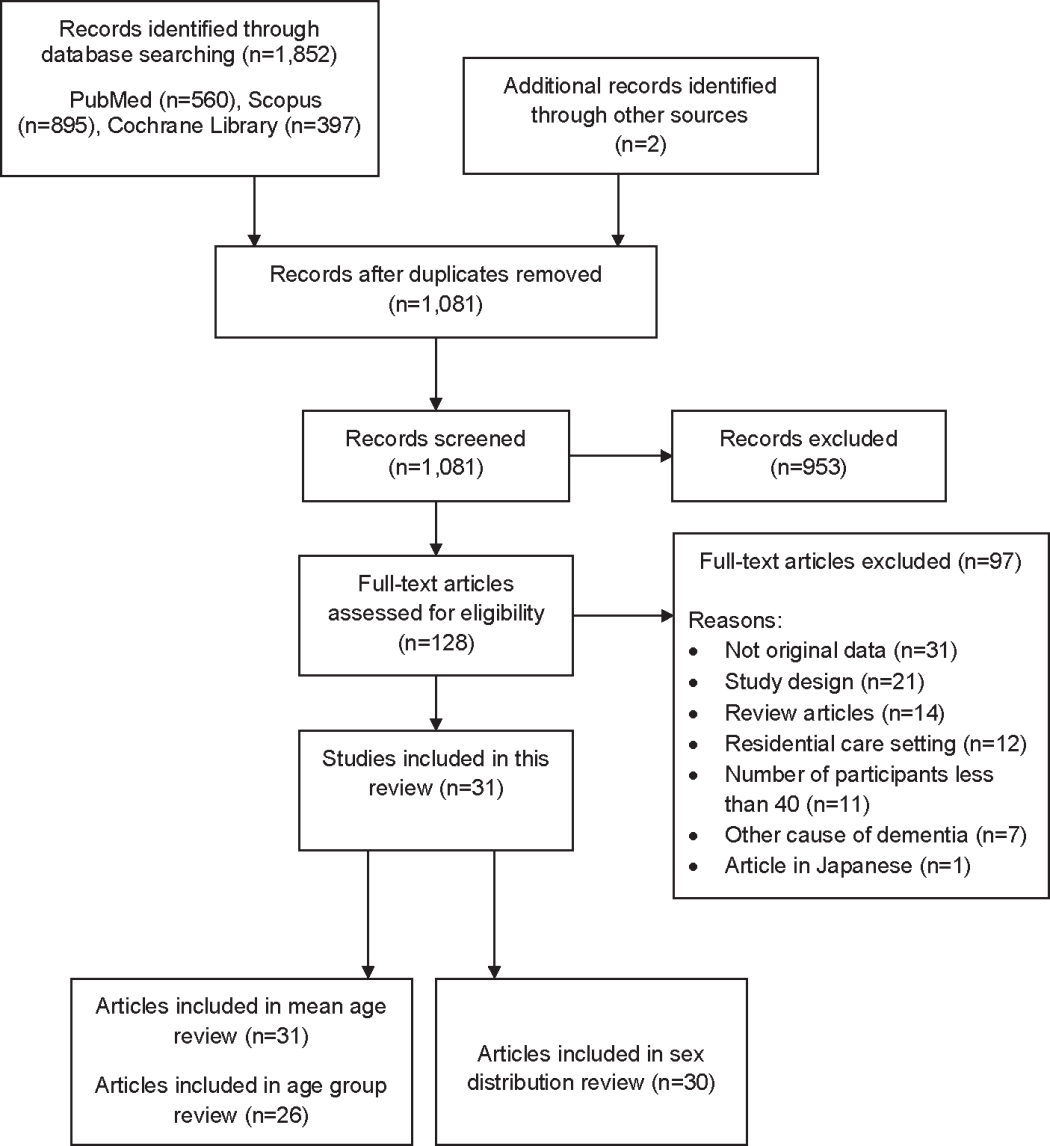
PRISMA (2009) flow diagram of article selection.

Altogether 1,852 citations were identified through the database search (Fig 1). Two articles were found via manual search of the reference lists. After removal of duplicates (n = 772), the title and abstract of each citation (n = 1,081) were screened by one author (AL) using the predefined selection criteria. All possibly relevant articles (n = 128) were retrieved for full text review. In obvious cases, articles were assessed by one author (AL), others by the whole team (SH, MK, AL). There were no disagreements after discussions and the decisions were made in consensus. Reference lists of the selected articles were searched for additional relevant publications. Based on full-text review, 97 articles were excluded and the reasons for exclusions are listed in S2 Appendix. Data extraction from the included articles (n = 31) considered data related to age (i.e. mean, standard deviation (SD) or standard error of the mean (SEM), range), sex and Mini-Mental State Examination Score (MMSE) [17] at the baseline of the double-blind treatment phase. In addition, data of comorbidities and use of concomitant drugs, inclusion and exclusion criteria, especially use of any age limits as selection criteria were collected. Supplementary data of articles (n = 5) were also searched from the electronic publications in order to find any additional information related to these aspects.

### Nationwide reference population with Alzheimer’s disease

We used data from a nationwide cohort of persons with a clinically verified diagnosis of Alzheimer’s disease [18] as a reference population. MEDication use among persons with ALZheimer’s disease (MEDALZ-2005) cohort includes all community-dwelling persons with AD in Finland in 2005 (n = 28,093).

More detailed description of the MEDALZ-2005 cohort has been published previously [18]. Briefly, information on AD diagnosis was received from the Finnish Special Reimbursement Register which contains information about the persons who are entitled to receive higher reimbursement of medication costs for specific chronic conditions such as diabetes, cardiovascular diseases and AD [18]. Data on all persons with reimbursement for AD medication on December 31, 2005 was extracted. The majority (over 90%) of AD cases were diagnosed in 2001–2005.

The Finnish Current Care Guideline on memory disorders recommends that all patients with AD should be prescribed AD medication unless there is a specific contraindication [19,20]. To be entitled for reimbursed AD medication, patient’s physician needs to send a medical statement to the Finnish Social Insurance Institution (SII) [18]. The SII requires that the medical statement verifies that the patient has: (a) symptoms consistent with AD; (b) experienced a decrease in social capacity over a period of at least 3 months; (c) received a computed tomography/magnetic resonance imaging scan; (d) had possible alternative diagnoses excluded; and (e) received confirmation of the diagnosis by a registered geriatrician or neurologist. The SII reviews all medical statements and checks that diagnosis of AD is based on the NINCDS-ADRDA and the DSM-IV criteria for Alzheimer’s disease [21,22]. The reimbursement for AD medication is granted for AD and dementia with features of AD and the cohort includes persons with mild to severe stages of AD.

The Finnish AD cohort is not selected on the basis of socioeconomic position as the Finnish public healthcare system is tax-funded covering all residents and guarantees equal access to these services regardless of age, region or income. According to the Finnish legislation, ethics committee approval was not required as only de-identified data were used in this study.

### Data analysis

From the included studies, the overall mean age and MMSE score were reported as weighted mean taking into account the number of participants in each study. The overall number of female participants was also counted. We calculated 95% confidence intervals (CI) for mean age (when possible) and proportion of female participants for each study. If age distribution of the study population was not reported in the article, we estimated the proportion of participants aged less than 65 years and participants aged over 75 years and over 85 years. The estimation was done using mean age, SD, range and the standard normal distribution assumption. In studies where age range or age limit was reported, we truncated normal distribution by the maximum likelihood method [23] using the lower and/or upper age range. If required information (i.e. mean age, SD or SEM) was not reported in the article, the study population was not included in the calculations (Table 2). Finally, we compared the proportions of participants in each age group in RCTs and in the reference AD population. To assess the differences in the mean age between these two data, one-sample t-test was used. The chi-square test was used to assess differences in sex and age distributions. Calculations were carried out using SPSS software version 21.0 (SPSS Inc., Chicago, IL, USA) and R software version 3.0.2 (R Foundation for Statistical Computing, Vienna, Austria, www.r-project.org).

**Table 2:** Baseline characteristics of study participants included into randomized controlled trials.

Author, year	Number of participants	Mean age in years (SD)	Age range	Age as inclusion criteria	Sex (female %)	Mean MMSE score
Rogers, 1996[24]	161	71.8 (NA)	54–85		60.2	18.6
Sramek, 1996[25]	50	68.0 (NA)	45–90		56.0	NA
Agid, 1998[26]	402/357^a^/386^a^	69.4 (8.4)^b^	50–90		56.2	NA
Corey-Bloom, 1998[27]	699	74.5 (NA)	45–89		60.9	19.7
Rogers, 1998[28]	468	73.7 (8.2)^b^	50–94	≥50	63.5	19.5
Rogers, 1998[29]	473	73.4 (7.5)^b^	51–94	≥50	61.9	19
Burns, 1999[30]	818	71.7 (8.3)^b^	50–93	≥50	57.5	20
Forette, 1999[31]	114/70^a^	71.2 (7.5)	NA		NA	19.5
Rösler, 1999[32]	725	72.0 (NA)	45–95	50–85	59.0	NA
Greenberg, 2000[33]	60	75.0 (9.5)	NA		50.0	21.8
Homma, 2000[34]	268/228^a^	69.8 (8.2)^b^	48–90		67.1	17.2
Raskind, 2000[35]	636	75.4 (8.3)^b^	NA		61.9	19.3
Tariot, 2000[36]	978	76.9 (7.7)^b^	NA		63.9	17.8
Wilcock, 2000[37]	653	72.2 (8.2)^b^	NA		62.6	19.3
Feldman, 2001[38]	290	73.6 (NA)	48–92		61.0	11.8
Mohs, 2001[39]	431	75.3 (8.8)^b^	49–94		62.9	17.1
Rockwood, 2001[40]	386	75.0 (7.4)^b^	NA		55.7	19.7
Wilkinson, 2001[41]	285	73.7 (8.2)^b^	NA	>45	57.5	18.7
Winblad, 2001[42]	286	72.5 (8.3)^b^	49–88	40–90	64.3	19.3
Krishnan, 2003[43]	67	73.4 (8.6)	NA	≥50	71.6	19.3
Lopez-Pousa, 2004[44]	218	77.6 (NA)	57–92	≥55	77.1	8.9
Seltzer, 2004[45]	153	74.0 (9.3)^b^	50–92		53.6	24.2
Brodaty, 2005[46]	971/965^a^	76.5 (7.8)	48–93		64.0	18.0
Karaman, 2005[47]	44	73.8 (4.1)^b^	NA	60–90	54.5	12.2
Rockwood, 2006[48]	130	77.5 (8.0)^b^	51–94		63.1	20.3
Black, 2007[49]	343	78.0 (8.1)	NA	≥50	70.3	7.6
Feldman, 2007[50]	678	71.4 (8.3)^b^	NA	≥50	59.0	18.6
Winblad, 2007[51]	1,195/1,190^a^	73.6 (7.8)^b^	NA	50–85	66.6	16.5
Homma, 2008[52]	302/290^a^	78.2 (8.1)^b^	NA	≥50	80.3	7.8
Nakamura, 2011[53]	859/855^a^	74.6 (7.2)	NA	50–85	68.3	16.6
Hager, 2014[54]	2045	73.0 (8.8)	NA	45–90	64.8	19.0
**Overall**	**15,032**	**73.9** ^**c**^			**63.2** ^**c**^	**17.8** ^**c**^

NA = information not available.

^a^Used in the calculations, not equal with the amount of randomized participants at baseline.

^b^SD for total population not reported in the study, it was calculated from reported SDs of treatment and placebo groups.

^c^Weighted mean by study sample size.

## Results

### Description of included studies

We found 31 original articles fulfilling our selection criteria; one of them was an imaging study [43]. Thirteen studies examined donepezil [24,28–30,33,34,38,39,42,43,45,49,52], ten rivastigmine [25–27,31,32,44,47,50,51,53] and eight galantamine [35–37,40,41,46,48,54]. Articles were published between the years 1996 and 2014. The majority of the studies (n = 28) were conducted in Europe or in North-America, and three studies in Japan. The diagnosis of AD was made in 28 studies by exclusion of other dementing disorders in accordance with the NINCDS-ADRDA criteria [21]. In three studies [34,46,52], diagnosis was made by using the DSM-IV criteria [22].

Altogether, 15,032 participants were included in reviewed RCTs (Table 2). The number of participants varied from 44 [47] to 2045 [54] and the median sample size was 357 persons. Duration of the treatment period varied from 9 weeks [25] to 2 years [54], the mean duration was 25.8 weeks. The range of MMSE score varied in the individual studies from 1 to 26, and the weighted overall mean was 17.8. In seven studies [26,31,34,46,51–53] the baseline characteristics such as mean age and sex distribution were not reported for all randomized participants.

Age as inclusion criteria was specified in 15 studies (48%) (Table 2) [28–30,32,41–44,47,49–54]. Both lower and upper age limit were used in six studies [32,42,47,51,53,54]. Age of at least 50 years was an inclusion criteria in ten studies [28–30,32,43,49–53] and other lower age limits were age of 40 [42], 45 [54], 46 [41], 55 [44] or 60 [47] years. The upper age criteria was either 85 [32,51,53] or 90 years [42,47,54].

### Age and sex distributions

**Fig 2:**
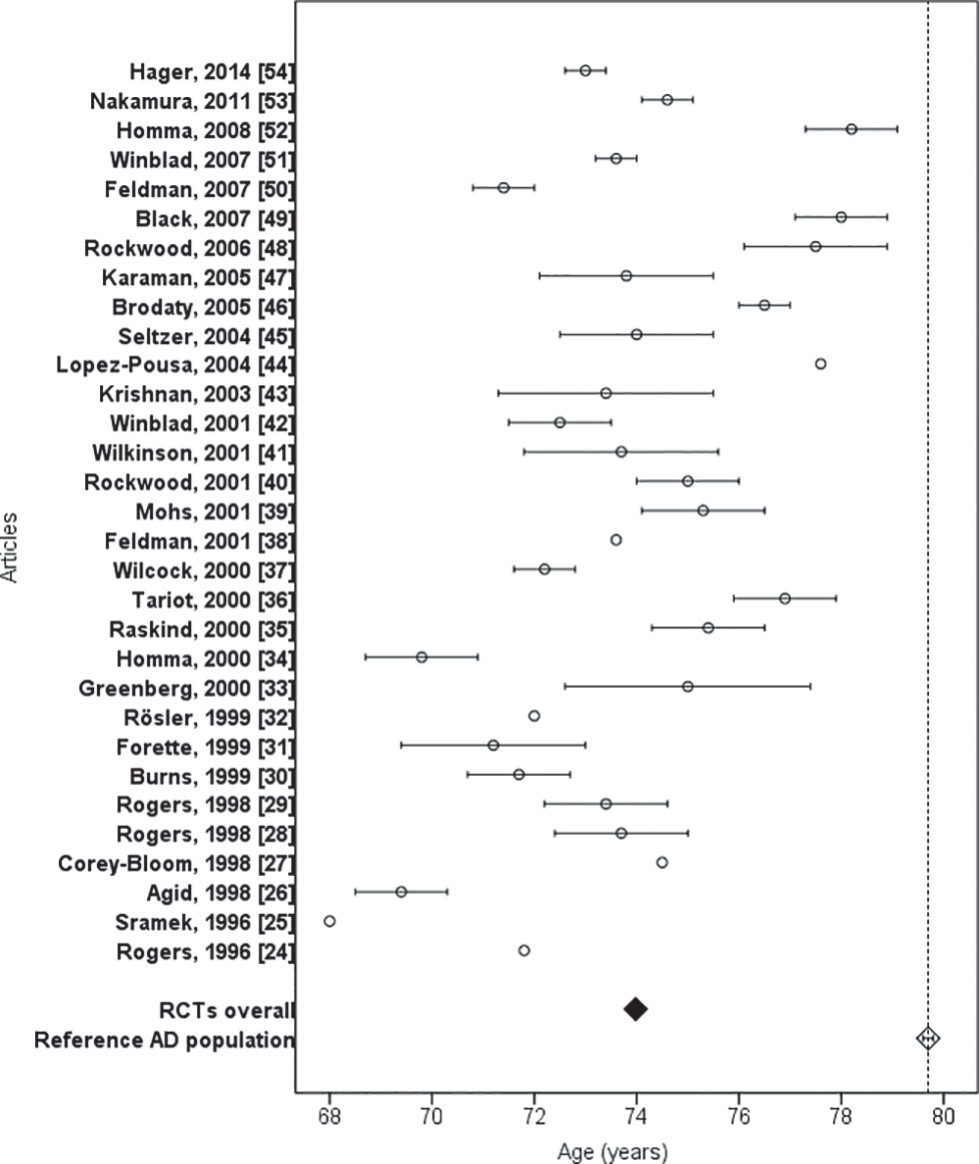
Mean age of RCT participants and reference population with Alzheimer’s disease.

The age range of participants included in RCTs was reported in 16 articles (52%) (Table 2), in these studies the lowest age of participants was 45 and highest 95 years. Mean age was reported in all articles and the overall weighted mean age of RCT participants was 73.9 years (Fig 2). Study population in RCTs was 5.8 years younger (95% CI 5.7 to 5.9, P < 0.001) compared with reference AD population. Mean age 79.7 years of real-life population with AD was not reached by any RCT.

**Fig 3:**
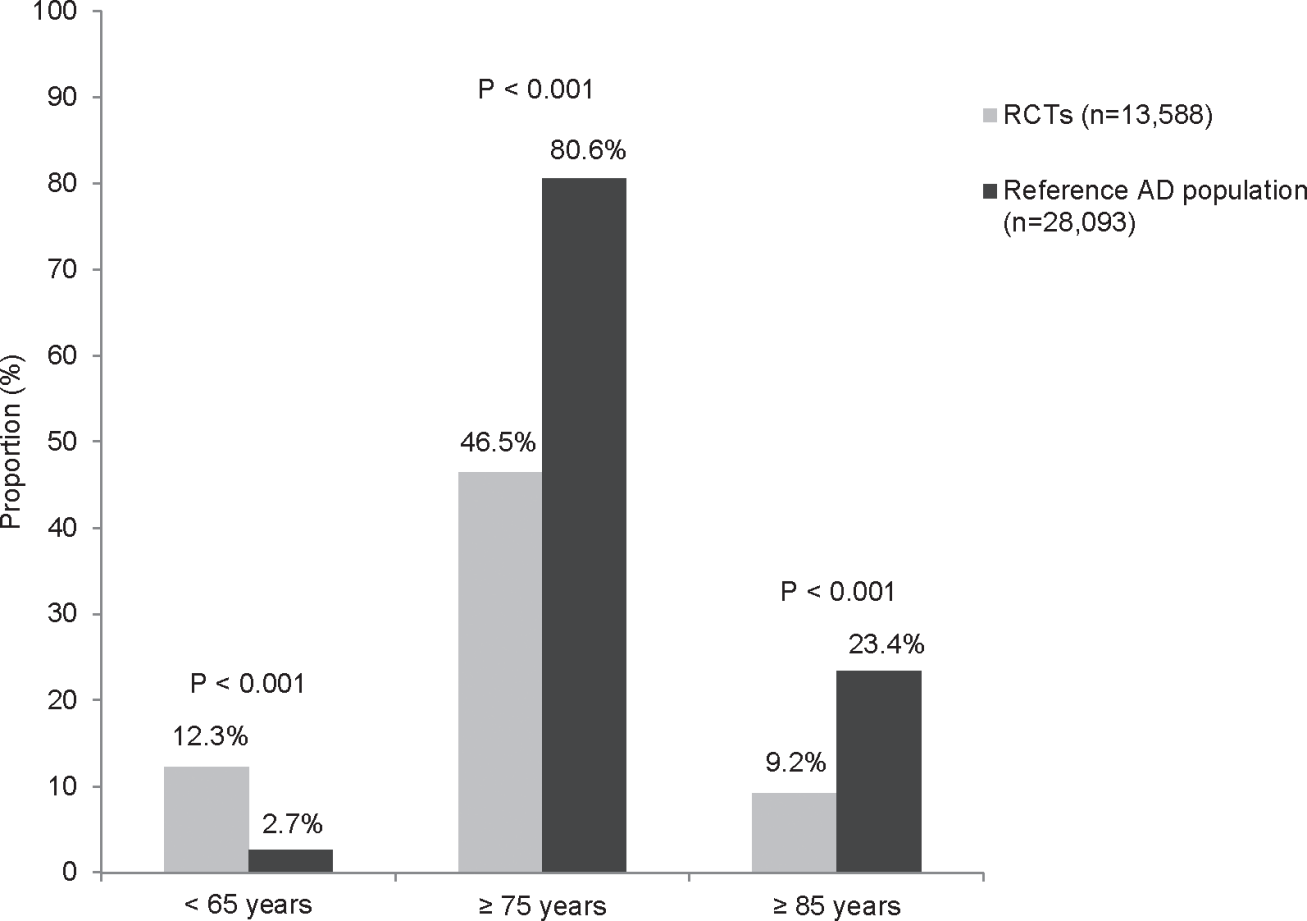
Proportion of persons aged <65 years, ≥75 years and ≥85 years in RCTs and reference population with Alzheimer’s disease.

Only four RCTs reported the age distribution of study participants [27,46,49,54]. Proportion of participants aged at least 75 years varied between RCTs from 26% [26] to 71% [49]. The difference in proportion of persons aged at least 75 years was 34.1% (46.5% vs. 80.6%) between RCTs and reference AD population (Fig 3). The proportion of participants aged at least 85 years varied from 0.3% [47] to 20.0% [52] in RCTs. The overall proportion of the oldest old participants in RCTs was less than half of the observed proportion (9.2% vs. 23.4%) in the reference AD population. Further, the overall proportion of participants aged less than 65 years was over four times higher in RCTs (12.3%) compared to the reference AD population (2.7%).

**Fig 4:**
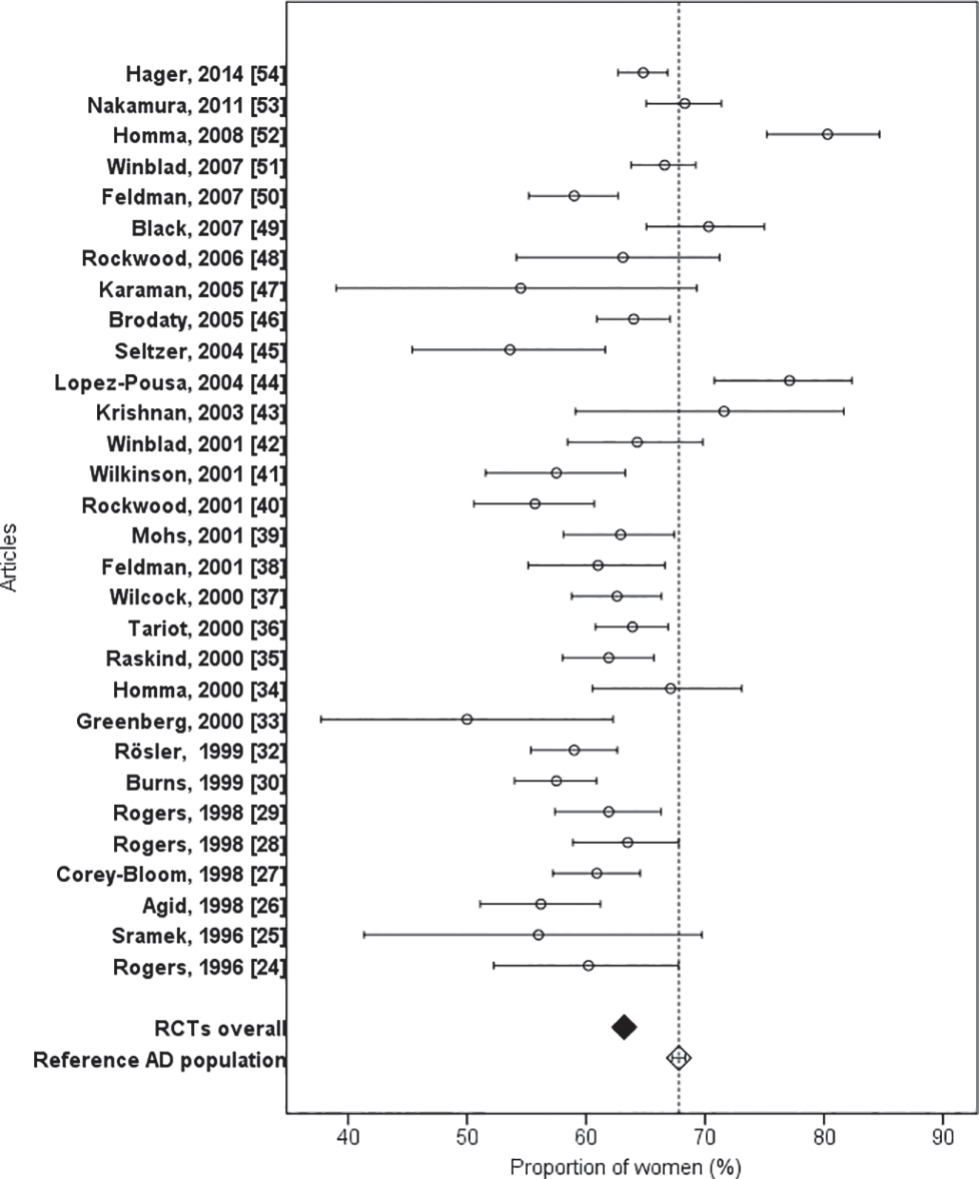
Proportion of women in RCTs and reference population with Alzheimer’s disease.

The proportion of female participants varied between 50.0% [33] to 80.3% [52], and sex distribution was reported in all articles except one [31] (Fig 4). The overall proportion of female participants in RCTs was 63.2% (n = 9,475/14,991) compared to 67.8% (n = 19,043/28,093) (P < 0.001) in the reference AD population. In five studies [43,44,49,52,53], the proportion of female participants was higher than in the reference AD population.

### Comorbidities and concomitant drug use

The number of exclusion criteria associated with comorbidities and concomitant drug use varied between studies from 0 [26] to 17 [28,38] and the mean number was 9.6. Most frequently exclusion criteria were related to psychiatric disorders in 22 studies [24,25,28,30,31,34–40,42–47,49,52–54] and cardiovascular diseases in 20 studies [24,25,28,29,32,35–38,40,42–44,46,47,49,50,52–54]. Insulin dependent diabetes was exclusion criteria in 12 studies [24,27–29,32,35–37,40–42,50]. Participants with stabile medical conditions such as hypertension or pulmonary disease were included and they were allowed to continue concomitant drugs for coexisting conditions [26,27,33,35–38,40,43,50,51,54]. Use of psychotropic or anticholinergic drugs were denied or dose was limited in 20 studies [25,27–29,32,34–36,38–43,49–54].

Both comorbid conditions and use of concomitant drugs were reported in ten articles (32%) [27,32,33,36–38,42,48,50,54]. Information on comorbid conditions was missing in 20 articles (65%). Most commonly reported comorbid conditions were cardiovascular and musculoskeletal diseases. Information on concomitant drugs use was reported in 14 articles (45%) [27,28,32,33,36–38,40,42,44,49,50,52,54] at the baseline and/or during the study, and the proportion of participants taking at least one concomitant drug varied from 79% to 97%. In the reference AD population, 95% used at least one concomitant drug other than AChEIs and memantine in the year 2005. The most frequently used groups were cardiovascular (73%) and psychotropic drugs (56%).

## Discussion

We found that participants in RCTs of acetylcholinesterase inhibitors were significantly younger than real-life AD population. There were considerable differences in the proportion of persons aged at least 75 years (one third lower) and especially in age group of 85 years or over where the proportion of the oldest old in RCTs was less than half of that in the real-life AD population. In addition, age distribution of study participants was poorly reported. It was positive to find out that the sex difference was small compared to age differences. However, comorbid conditions and use of concomitant drugs were rarely reported in the articles, so we were unable to compare health conditions between RCT participants and nationwide reference AD population.

Our findings about the age gap are in line with previous studies reporting that participants included in clinical trials of dementia are significantly younger than the real-life population with dementia [55,56]. Underrepresentation of older people has also been identified in RCTs concerning cardiovascular diseases [2,6,7], depression [8] and cancer [4,5]. After 1990, enrollment of older people improved in clinical trials of acute coronary syndromes but still more than half of the studies did not enroll participants aged 75 years or older in trials published between years 1996–2000 [6]. In our review, we found that the oldest old participants (at least 85 years) were underrepresented and the proportion of younger participants (under 65 years of age) was over four times greater in RCTs than in reference AD population. In addition, the age distribution of participants was lacking in 87% of articles. Our findings are alarming since already in the year 1994 ICH guideline on geriatrics (E7) [13] was adopted by the regulatory authorities in Europe, Japan and US. According to the guidelines, it is important to include older people into the clinical trials, if they are likely to be treated with the drug in question or condition is associated with aging like Alzheimer’s disease [11,13].

Removal of upper age limit from the inclusion/exclusion criteria alone does not improve representation of older people, because comorbidities and concomitant drugs are also important factors for exclusion of older people from the clinical trials [57,58]. In addition, cognitive impairment and capacity to consent personally to research may restrict participation to clinical trials [59]. Whether comorbidities and/or concomitant drugs were reasons for exclusion of the oldest old from RCTs of acetylcholinesterase inhibitors was impossible to extract from the published studies, because reasons for exclusion during recruitment were not reported. Comorbidities and/or concomitant drugs use may explain the lack of oldest old in the reviewed studies because a previous review found that more than half of the older people receiving donepezil would have been ineligible to participate in donepezil RCTs due to comorbidities and concomitant drugs [56].

In previous studies women have been found to be underrepresented in RCTs [7,60,61]. Thus, it was a positive finding that the proportion of female participants included in RCTs of acetylcholinesterase inhibitors was near real-life patient population with AD.

### Strengths and limitations of the study

The strength of this review was ability to compare the RCT participants to nationwide population with clinically verified AD. The reference population with Alzheimer’s disease represents a real-life community-dwelling user population of AChEIs in Finland. We are not aware of any other cohort covering persons with clinically and imaging verified diagnoses of AD from the whole country. In addition, the Finnish reference cohort is not selected by socioeconomic status as the Finnish tax-funded healthcare system covers all residents. In Finland, AChEI use is recommended for all patients with Alzheimer’s disease if there are no contraindications for use. The guidance and reimbursement of AChEI use may be more restricted in other countries which could affect the age and gender distribution of AChEI users. However, a large population-based healthcare administrative database study[62] describing the use of AChEIs from Ontario, Canada (n = 28,961) supports our findings of underrepresentation of older people in RCTs. The Canadian study was based on the Ontario Drug Benefit claims data that covers AChEIs for patients aged over 65 years with a probable AD diagnosis and MMSE score between 10 and 26. The mean age of new AChEI users between 2000 and 2002 was 80.3 and 63.4% were female. In addition, article describing a large French National Alzheimer database reported that the mean age of 90,176 patients with Alzheimer’s disease recorded in the database between 2009 and 2012 was 81.9 years, 70.6% of patients were female and mean MMSE score was 16.4 [63]. The French database records only visits of patients consulting specialized memory units and independent specialist for memory diseases which can affect the age and gender distribution of the recorded patients with AD. Although these two cohorts [62,63] are more restricted than the Finnish AD cohort used in this review as the reference population, both databases strengthen our findings that RCT participants are younger than the real-life user population with AD and the majority of RCT participants should be female.

Due to the lack of detailed information on participants’ age distribution in RCTs, we had to use normal distribution assumption to estimate the proportion of participants in each age group. We used age range or age as inclusion criteria to truncate normal distribution in order to obtain as precise estimates of proportions as possible. However, we cannot exclude possibility that some studies differ from this assumption and proportions can be over- or underestimated.

### Meaning of the study

To our knowledge, this is the first systematic review including RCTs of all three AChEIs and where data of age and sex distribution were compared with a real-life user population of AChEIs. It is important to ensure evidence based medicine to older people, also for those aged 80 or over, which is one of the fastest growing age group in the European population [1]. Several steps have already been taken such as guidelines and recommendations have been published in order to improve representation of older people in clinical trials [11,13,14]. In addition, ethics committees have an important role in reducing unjustified exclusion by age, comorbidities and concomitant drugs while they are reviewing and approving clinical trial applications. Editors and peer-reviewers of journals have an opportunity to pay more attention that new articles follow the guidelines in relation to adequately representing the target patient population. They should also require authors to report detailed age distribution of participants and describe prevalence of comorbidities and use of concomitant drugs. Reporting of these factors would enable comparison to real-life users and help to reduce bias of studying non-representative populations.

## Conclusions

There is a discrepancy in age distribution between RCTs of acetylcholinesterase inhibitors and real-life AD population which needs to be considered when designing future clinical trials. Further, more detailed reporting of age distribution, comorbidities and concomitant drugs would be important information for clinicians in evaluating conclusions from RCTs to real-life practice. The existing recommendations of inclusion of older people should be followed to ensure safe pharmacotherapy for older people.

## Supporting Information

S1 Checklist (DOCX)

S1 Appendix Full electronic search strategy for identification of RCTs of donepezil, rivastigmine and galantamine from Scopus database. (DOCX)

S2 Appendix Full-text articles excluded from the review. (DOCX)
