# Supplementary material for: Systematic Review: Representativeness of Participants in RCTs of Acetylcholinesterase Inhibitors
Source: PLoS One. 2015 May 1;10(5):e0124500. doi: 10.1371/journal.pone.0124500 (PMC4416896; doi:10.1371/journal.pone.0124500)
Supplement: S1 Appendix — (DOCX) [file pone.0124500.s002.docx]

**S1 Appendix Full electronic search strategy for identification of RCTs of donepezil, rivastigmine and galantamine from Scopus database**

**Publication date:** from inception to January 4, 2015

**Limits used:** none

**Document type:** all

**Search strategy to identify RCTs of donepezil** (411 search results):

(alzheimer disease OR alzheimer*)

AND ("randomi* controlled trial*" OR (random* W/3 allocat*) OR randomi* OR double-blind method OR "double blind")

AND (placebo*)

AND (donepezil OR aricept OR "E 2020" OR "E-2020" OR e2020)

**Search strategy to identify RCT s of rivastigmine** (258 search results):

(alzheimer disease OR alzheimer*)

AND ("randomi* controlled trial*" OR (random* W/3 allocat*) OR randomi* OR double-blind method OR "double blind")

AND (placebo*)

AND (rivastigmine OR exelon OR ena OR "SDZ ENA 713")

**Search strategy to identify RCTs of galantamine** (226 search results):

(alzheimer disease OR alzheimer*)

AND ("randomi* controlled trial*" OR (random* W/3 allocat*) OR randomi* OR double-blind method OR "double blind")

AND (placebo*)

AND (galantamin* OR galanthamin* OR reminyl)

**Altogether, searches in Scopus retrieved 895 citations.**
