# Supplementary material for: Systematic Review: Representativeness of Participants in RCTs of Acetylcholinesterase Inhibitors
Source: PLoS One. 2015 May 1;10(5):e0124500. doi: 10.1371/journal.pone.0124500 (PMC4416896; doi:10.1371/journal.pone.0124500)
Supplement: S2 Appendix — (DOCX) [file pone.0124500.s003.docx]

**S2 Appendix: Full-text articles excluded from the review**

This table includes only one primary reason for exclusion for each article. However, there could have been multiple reasons for the exclusion.

| **First author and date** | **Reason** |
| --- | --- |
| Rainer, 1997 [1] | Review article |
| Homma, 1998 [2] | Article in Japanese |
| Rogers, 1998 [3] | Review article |
| Rogers, 1998 [4] | Not original data  Original data included in this review: Rogers, 1998 [Ref. number in the manuscript 28] |
| Rogers, 1998 [5] | Study design |
| Anand, 2000 [6] | Not original data (pooled data) |
| Farlow, 2000 [7] | Study design |
| Knopman, 2000 [8] | Review article |
| Kumar, 2000 [9] | Not original data  Original data included in this review: Corey-Bloom, 1998 [Ref. number in the manuscript 27] |
| Potkin, 2000 [10] | Number of participants under 40 |
| Nakano, 2001 [11] | Number of participants under 40 |
| Tariot, 2001 [12] | Residential care setting |
| Tariot, 2001 [13] | Review article |
| Farlow, 2001 [14] | Not original data  Original data included in this review: Corey-Bloom, 1998 [Ref. number in the manuscript 27] |
| Doraiswamy, 2002 [15] | Review article |
| Erkinjuntti, 2002 [16] | Not original data  Original data included in this review: Rösler, 1999 [Ref. number in the manuscript 32] |
| Erkinjuntti, 2002 [17] | Other cause of dementia |
| Gauthier, 2002 [18] | Not original data (sub-analysis)  Original data included in this review: Feldman, 2001 [Ref. number in the manuscript 38] |
| Gauthier, 2002 [19] | Not original data  Original data included in this review: Feldman, 2001 [Ref. number in the manuscript 38] |
| Lilienfeld, 2002 [20] | Other cause of dementia |
| Mori, 2002 [21] | Review article |
| Pratt, 2002 [22] | Review article |
| Wilkinson, 2002 [23] | Not original data (post-hoc analysis of pooled data) |
| Erkinjuntti, 2003 [24] | Study design |
| Farlow, 2003 [25] | Review article |
| Feldman, 2003 [26] | Not original data  Original data included in this review: Feldman, 2001 [Ref. number in the manuscript 38] |
| Finucane, 2003 [27] | Residential care setting |
| Geldmacher, 2003 [28] | Review article |
| Kozubski, 2003 [29] | Study design |
| Kemp, 2003 [30] | Number of participants under 40 |
| Kurz, 2003 [31] | Other cause of dementia |
| Marcusson, 2003 [32] | Not original data (post-hoc analysis of pooled data) |
| Markowitz, 2003 [33] | Not original data  Original data included in this review: Rockwood, 2001 [Ref. number in the manuscript 40] |
| Tariot, 2003 [34] | Residential care setting |
| Tune, 2003 [35] | Number of participants under 40 |
| Wimo, 2003 [36] | Not original data  Original data included in this review: Winblad, 2001 [Ref. number in the manuscript 42] |
| Bullock, 2004[37] | Other cause of dementia |
| Burns, 2004 [38] | Not original data (pooled data) |
| Courtney, 2004 [39] | Study design |
| Cummings, 2004 [40] | Not original data  Original data included in this review: Tariot, 2000 [Ref. number in the manuscript 36] |
| Emre, 2004 [41] | Other cause of dementia |
| Feldman, 2004 [42] | Not original data  Original data included in this review: Feldman, 2001 [Ref. number in the manuscript 38] |
| Gauthier, 2004 [43] | Not original data  Original data included in this review: Feldman, 2001 [Ref. number in the manuscript 38] |
| Grossberg, 2004 [44] | Study design |
| Holmes, 2004 [45] | Study design |
| Lyketsos, 2004 [46] | Study design |
| Johannsen, 2004 [47] | Review article |
| Raskind, 2004 [48] | Study design |
| Thompson, 2004 [49] | Review article |
| Dujardin, 2006 [50] | Other cause of dementia |
| Johannsen, 2006 [51] | Study design |
| Marder, 2006 [52] | Residential care setting |
| Moraes, 2006 [53] | Number of participants under 40 |
| Robinson, 2006 [54] | Not original data (post-hoc analyses)  Original data included in this review: Brodaty, 2005 [Ref. number in the manuscript 46]. |
| Teipel, 2006 [55] | Number of participants under 40 |
| Winblad, 2006 [56] | Study design |
| Winblad, 2006 [57] | Residential care setting |
| Burns, 2007 [58] | Study design |
| Howard, 2007 [59] | Residential care setting |
| Mahlberg, 2007 [60] | Residential care setting |
| Winblad, 2007 [61] | Not original data  Original data included in this review: Winblad, 2007 [Ref. number in the manuscript 51]. |
| Winblad, 2007 [62] | Not original data  Original data included in this review: Winblad, 2007 [Ref. number in the manuscript 51]. |
| Winstein, 2007 [63] | Number of participants under 40 |
| Darreh-Shori, 2008 [64] | Number of participants under 40 |
| Dichgans, 2008 [65] | Other cause of dementia |
| Doody, 2008 [66] | Study design |
| Erkinjuntti, 2008 [67] | Not original data  Reports same data as excluded study Erkinjuntti 2002 [17]. |
| Jelic, 2008 [68] | Residential care setting |
| Kadir, 2008 [69] | Not original data  Reports same data as excluded study Darreh-Shori 2008 [64]. |
| Moraes, 2008 [70] | Number of participants under 40 |
| Aronson, 2009 [71] | Not original data (post-hoc analysis)  Original data included in this review: Tariot, 2000 [Ref. number in the manuscript 36]. |
| Ballard, 2009 [72] | Residential care setting |
| Burns, 2009 [73] | Residential care setting |
| Grossberg, 2009 [74] | Study design |
| Cummings, 2010 [75] | Not original data (analysis of RCT and open label extension)  Original data from RCT included in this review: Winblad, 2007 [Ref. number in the manuscript 51]. |
| Farlow, 2010 [76] | Not original data (pooled data) |
| Farlow, 2010 [77] | Study design |
| Gauthier, 2010 [78] | Not original data (pooled data) |
| Jelic, 2010 [79] | Review article |
| Seltzer, 2010 [80] | Review article |
| Alva, 2011 [81] | Not original data (post-hoc analysis) |
| Burns, 2011 [82] | Residential care setting |
| Farlow, 2011 [83] | Not original data (retrospective analysis)  Original data included in this review: Winblad, 2007 [Ref. number in the manuscript 51]. |
| Gaudig, 2011 [84] | Study design |
| Grossberg, 2011 [85] | Not original data (post-hoc analysis) |
| Keller, 2011 [86] | Not original data  Reports same data as excluded study as Darreh-Shori 2008 [64]. |
| Miettinen, 2011 [87] | Number of participants under 40 |
| Scarpini, 2011 [88] | Study design |
| Andersen, 2012 [89] | Residential care setting |
| Likitjaroen, 2012 [90] | Number of participants under 40 |
| McLaren, 2012 [91] | Study design |
| Farlow, 2013 [92] | Study design |
| Ferris, 2013 [93] | Not original data  Original data included in this review: Lopez-Pousa, 2004 [Ref. number in the manuscript 44]. |
| Rountree, 2013 [94] | Review article |
| Sole-Padulles, 2013 [95] | Study design |
| Ohnishi, 2014 [96] | Not original data (post-hoc analysis)  Original data reported in Japanese |
| Pelton, 2014 [97] | Study design |

**References**

1. Rainer M. Clinical studies with galanthamine. Drugs of Today. 1997;33: 273-279.

2. Homma A, Imai Y, Hariguchi S, Hasegawa K, Kameyama M, Nishimura T. Late phase II clinical study of acetylcholinesterase inhibitor E 2020 in patients with Alzheimer-type dementia - 12-weeks double-blind, placebo-controlled study 3 mg/day, 5mg/day. Clinical Evaluation. 1998;26: 251-284.

3. Rogers SL. Perspectives in the management of Alzheimer's disease: Clinical profile of donepezil. Dement Geriatr Cogn Disord. 1998;9 Suppl 3: 29-42.

4. Rogers SL, Farlow MR, Doody RS, Mohs R, Friedhoff LT, Ieni J, et al. Donepezil improved cognitive and global function in mild-to-moderate Alzheimer disease. Evidence-Based Medicine. 1998;3: 155.

5. Rogers SL, Friedhoff LT. Long-term efficacy and safety of donepezil in the treatment of Alzheimer's disease: An interim analysis of the results of a US multicentre open label extension study. European Neuropsychopharmacology. 1998;8: 67-75.

6. Anand R, Messina J, Hartman R. Dose-response effect of rivastigmine in the treatment of Azheimer's disease. Int J Geriatr Psychopharmacol. 2000;2: 68-72.

7. Farlow M, Anand R, Messina Jr. J, Hartman R, Veach J. A 52-week study of the efficacy of rivastigmine in patients with mild to moderately severe Alzheimer's disease. Eur Neurol. 2000;44: 236-241.

8. Knopman DS. Management of cognition and function: New results from the clinical trials programme of Aricept® (donepezil HCl). Int J Neuropsychopharmacol. 2000;3: S13-S20.

9. Kumar V, Anand R, Messina J, Hartman R, Veach J. An efficacy and safety analysis of Exelon® in Alzheimer's disease patients with concurrent vascular risk factors. Eur J Neurol. 2000;7: 159-169.

10. Potkin SG, Anand R, Fleming K, Alva G, Keator D, Carreon D, et al. Brain metabolic and clinical effects of rivastigmine in Alzheimer's disease. Int J Neuropsychopharmacol. 2001;4: 223-230.

11. Nakano S, Asada T, Matsuda H, Uno M, Takasaki M. Donepezil hydrochloride preserves regional cerebral blood flow in patients with Alzheimer's disease. J Nucl Med. 2001;42: 1441-1445.

12. Tariot PN, Cummings JL, Katz IR, Mintzer J, Perdomo CA, Schwam EM, et al. A randomised, double-blind, placebo-controlled study of the efficacy and safety of donepezil in patients with Alzheimer's disease in the nursing home setting. J Am Geriatr Soc. 2001;49: 1590-1599.

13. Tariot PN. Maintaining cognitive function in Alzheimer disease: How effective are current treatments? Alzheimer Dis Assoc Disord. 2001;15 Suppl 1: S26-33.

14. Farlow MR, Hake A, Messina J, Hartman R, Veach J, Anand R. Response of patients with Alzheimer disease to rivastigmine treatment is predicted by the rate of disease progression. Arch Neurol. 2001;58: 417-422.

15. Doraiswamy PM, Krishnan KRR, Anand R, Sohn H, Danyluk J, Hartman RD, et al. Long-term effects of rivastigmine in moderately severe Alzheimer's disease: Does early initiation of therapy offer sustained benefits? Prog Neuro-Psychopharmacol Biol Psychiatry. 2002;26: 705-712.

16. Erkinjuntti T, Skoog I, Lane R, Andrews C. Rivastigmine in patients with Alzheimer's disease and concurrent hypertension. Int J Clin Pract. 2002;56: 791-796.

17. Erkinjuntti T, Kurz A, Gauthier S, Bullock R, Lilienfeld S, Damaraju CV. Efficacy of galantamine in probable vascular dementia and Alzheimer's disease combined with cerebrovascular disease: A randomised trial. Lancet. 2002;359: 1283-1290.

18. Gauthier S, Feldman H, Hecker J, Vellas B, Emir B, Subbiah P. Functional, cognitive and behavioral effects of donepezil in patients with moderate Alzheimer's disease. Curr Med Res Opin. 2002;18: 347-354.

19. Gauthier S, Feldman H, Hecker J, Vellas B, Ames D, Subbiah P, et al. Efficacy of donepezil on behavioral symptoms in patients with moderate to severe Alzheimer's disease. Int Psychogeriatr. 2002;14: 389-404.

20. Lilienfeld S, Kurz A. Broad therapeutic benefits in patients with probable vascular dementia or Alzheimer's disease with cerebrovascular disease treated with galantamine. Ann N Y Acad Sci. 2002;977: 487-492.

21. Mori S. Responses to donepezil in Alzheimer's disease and parkinson's disease. Ann N Y Acad Sci. 2002;977: 493-500.

22. Pratt RD, Perdomo CA, Surick IW, Ieni JR. Donepezil: Tolerability and safety in Alzheimer's disease. Int J Clin Pract. 2002;56: 710-717.

23. Wilkinson DG, Hock C, Farlow M, Van Baelen B, Schwalen S. Galantamine provides broad benefits in patients with 'advanced moderate' Alzheimer's disease (MMSE ≤12) for up to six months. Int J Clin Pract. 2002;56: 509-514.

24. Erkinjuntti T, Skoog I, Lane R, Andrews C. Potential long-term effects of rivastigmine on disease progression may be linked to drug effects on vascular changes in Alzheimer brains. Int J Clin Pract. 2003;57: 756-760.

25. Farlow M, Potkin S, Koumaras B, Veach J, Mirski D. Analysis of outcome in retrieved dropout patients in a rivastigmine vs placebo, 26-week, Alzheimer disease trial. Arch Neurol. 2003;60: 843-848.

26. Feldman H, Gauthier S, Hecker J, Vellas B, Emir B, Mastey V, et al. Efficacy of donepezil on maintenance of activities of daily living in patients with moderate to severe Alzheimer's disease and the effect on caregiver burden. J Am Geriatr Soc. 2003;51: 737-744.

27. Finucane TE, Tariot PN, Cummings JL, Katz IR, Mintzer J, Perdomo CA. Getting donepezil into the nursing home. A randomized, double-blind, placebo-controlled study of the efficacy and safety of donepezil in patients with Alzheimer's disease in the nursing home setting. J Am Geriatr Soc. 2003;51: 133-134.

28. Geldmacher DS, Provenzano G, McRae T, Mastey V, Ieni JR. Donepezil is associated with delayed nursing home placement in patients with Alzheimer's disease. J Am Geriatr Soc. 2003;51: 937-944.

29. Kozubski W, Hasselbalch S, Jakab G, Kalisvaart CJ, Kurz A, McCarthy J, et al. Donepezil-treated Alzheimer's disease patients with apparent initial cognitive decline demonstrate significant benefits when therapy is continued: Results from a randomized, placebo-controlled trial. Eur Neuropsychopharmacol. 2003;13: S405.

30. Kemp PM, Holmes C, Hoffmann S, Wilkinson S, Zivanovic M, Thom J, et al. A randomised placebo controlled study to assess the effects of cholinergic treatment on muscarinic receptors in Alzheimer's disease. J Neurol Neurosurg Psychiatry. 2003;74: 1567-1570.

31. Kurz AF, Erkinjuntti T, Small GW, Lilienfeld S, Venkata Damaraju CR. Long-term safety and cognitive effects of galantamine in the treatment of probable vascular dementia or Alzheimer's disease with cerebrovascular disease. Eur J Neurol. 2003;10: 633-640.

32. Marcusson J, Bullock R, Gauthier S, Kurz A, Schwalen S. Galantamine demonstrates efficacy and safety in elderly patients with Alzheimer disease. Alzheimer Dis Assoc Disord. 2003;17 Suppl 3: S86-91.

33. Markowitz JS, Gutterman EM, Lilienfeld S, Papadopoulos G. Sleep-related outcomes in persons with mild to moderate Alzheimer disease in a placebo-controlled trial of galantamine. Sleep. 2003;26: 602-606.

34. Tariot PN, Cummings JL, Katz IR, Mintzer J, Perdomo CA, Schwam EM. A randomized, double-blind, placebo-controlled study of the efficacy and safety of donepezil in patients with Alzheimer's disease in the nursing home setting J am geriatr soc 2001;49:1590-9. J Am Geriatr Soc. 2003;51: 133-134.

35. Tune L, Tiseo PJ, Ieni J, Perdomo C, Pratt RD, Votaw JR, et al. Donepezil HCl (E2020) maintains functional brain activity in patients with Alzheimer disease: Results of a 24-week, double-blind, placebo-controlled study. Am J Geriatr Psychiatry. 2003;11: 169-177.

36. Wimo A, Winblad B, Engedal K, Soininen H, Verhey F, Waldemar G, et al. An economic evaluation of donepezil in mild to moderate Alzheimer's disease: Results of a 1-year, double-blind, randomized trial. Dement Geriatr Cogn Disord. 2003;15: 44-54.

37. Bullock R, Erkinjuntti T, Lilienfeld S. Management of patients with Alzheimer's disease plus cerebrovascular disease: 12-month treatment with galantamine. Dement Geriatr Cogn Disord. 2004;17: 29-34.

38. Burns A, Spiegel R, Quarg P. Efficacy of rivastigmine in subjects with moderately severe Alzheimer's disease. Int J Geriatr Psychiatry. 2004;19: 243-249.

39. Courtney C, Farrell D, Gray R, Hills R, Lynch L, Sellwood E, et al. Long-term donepezil treatment in 565 patients with Alzheimer's disease (AD2000): Randomised double-blind trial. Lancet. 2004;363: 2105-2115.

40. Cummings JL, Schneider L, Tariot PN, Kershaw PR, Yuan W. Reduction of behavioral disturbances and caregiver distress by galantamine in patients with Alzheimer's disease. Am J Psychiatry. 2004;161: 532-538.

41. Emre M, Aarsland D, Albanese A, Byrne EJ, Deuschl G, Deyn PP, et al. Rivastigmine for dementia associated with Parkinson's disease. N Engl J Med. 2004;351: 2509-2518.

42. Feldman H, Gauthier S, Hecker J, Vellas B, Hux M, Xu Y, et al. Economic evaluation of donepezil in moderate to severe Alzheimer disease. Neurology. 2004;63: 644-650.

43. Gauthier S. Efficacy of donepezil on maintenance of activities of daily living in patients with moderate-to-severe Alzheimer's disease, and impact on caregiver burden. Geriatrics and Aging. 2004;7: 34-36.

44. Grossberg G, Irwin P, Satlin A, Mesenbrink P, Spiegel R. Rivastigmine in Alzheimer disease: Efficacy over two years. Am J Geriatr Psychiatry. 2004;12: 420-431.

45. Holmes C, Wilkinson D, Dean C, Vethanayagam S, Olivieri S, Langley A, et al. The efficacy of donepezil in the treatment of neuropsychiatric symptoms in Alzheimer disease. Neurology. 2004;63: 214-219.

46. Lyketsos CG, Reichman WE, Kershaw P, Zhu Y. Long-term outcomes of galantamine treatment in patients with Alzheimer disease. Am J Geriatr Psychiatry. 2004;12: 473-482.

47. Johannsen P. Long-term cholinesterase inhibitor treatment of Alzheimer's disease. CNS Drugs. 2004;18: 757-768.

48. Raskind MA, Peskind ER, Truyen L, Kershaw P, Damaraju CV. The cognitive benefits of galantamine are sustained for at least 36 months: A long-term extension trial. Arch Neurol. 2004;61: 252-256.

49. Thompson S, Lanctôt KL, Hermann N. The benefits and risks associated with cholinesterase inhibitor therapy in Alzheimer's disease. Expert Opinion on Drug Safety. 2004;3: 425-440.

50. Dujardin K, Devos D, Duhem S, Destée A, Marié R-, Durif F, et al. Utility of the mattis dementia rating scale to assess the efficacy of rivastigmine in dementia associated with parkinson's disease. J Neurol. 2006;253: 1154-1159.

51. Johannsen P, Salmon E, Hampel H, Xu Y, Richardson S, Qvitzau S, et al. Assessing therapeutic efficacy in a progressive disease: A study of donepezil in Alzheimer's disease. CNS Drugs. 2006;20: 311-325.

52. Marder K. Donepezil in patients with severe Alzheimer's disease: Double-blind parallel-group, placebo controlled study. Curr Neurol Neurosci Rep. 2006;6: 364-363.

53. Moraes Wdos S, Poyares DR, Guilleminault C, Ramos LR, Bertolucci PH, Tufik S. The effect of donepezil on sleep and REM sleep EEG in patients with Alzheimer disease: A double-blind placebo-controlled study. Sleep. 2006;29: 199-205.

54. Robinson DM, Plosker GL. Galantamine extended release. CNS Drugs. 2006;20: 673-81; discussion 682-3.

55. Teipel SJ, Drzezga A, Bartenstein P, Möller HJ, Schwaiger M, Hampel H. Effects of donepezil on cortical metabolic response to activation during 18FDG-PET in Alzheimer's disease: A double-blind cross-over trial. Psychopharmacology (Berl ). 2006;187: 86-94.

56. Winblad B, Wimo A, Engedal K, Soininen H, Verhey F, Waldemar G, et al. 3-year study of donepezil therapy in Alzheimer's disease: Effects of early and continuous therapy. Dement Geriatr Cogn Disord. 2006;21: 353-363.

57. Winblad B, Kilander L, Eriksson S, Minthon L, Batsman S, Wetterholm AL, et al. Donepezil in patients with severe Alzheimer's disease: Double-blind, parallel-group, placebo-controlled study. Lancet. 2006;367: 1057-1065.

58. Burns A, Gauthier S, Perdomo C. Efficacy and safety of donepezil over 3 years: An open-label, multicentre study in patients with Alzheimer's disease. Int J Geriatr Psychiatry. 2007;22: 806-812.

59. Howard RJ, Juszczak E, Ballard CG, Bentham P, Brown RG, Bullock R, et al. Donepezil for the treatment of agitation in Alzheimer's disease. N Engl J Med. 2007;357: 1382-1392.

60. Mahlberg R, Walther S, Eichmann U, Tracik F, Kunz D. Effects of rivastigmine on actigraphically monitored motor activity in severe agitation related to Alzheimer's disease: A placebo-controlled pilot study. Arch Gerontol Geriatr. 2007;45: 19-26.

61. Winblad B, Grossberg G, Frölich L, Farlow M, Zechner S, Nagel J, et al. IDEAL: A 6-month, double-blind, placebo-controlled study of the first skin patch for Alzheimer disease. Neurology. 2007;69: S14-S22.

62. Winblad B, Kawata AK, Beustrrieri KM, Thomas SK, Wimo A, Lane R, et al. Caregiver preference for rivastigmine patch relative to capsules for treatment of probable Alzheimer's disease. Int J Geriatr Psychiatry. 2007;22: 485-491.

63. Winstein CJ, Bentzen KR, Boyd L, Schneider LS. Does the cholinesterase inhibitor, donepezil, benefit both declarative and non-declarative processes in mild to moderate Alzheimer's disease? Curr Alzheimer Res. 2007;4: 273-276.

64. Darreh-Shori T, Kadir A, Almkvist O, Grut M, Wall A, Blomquist G, et al. Inhibition of acetylcholinesterase in CSF versus brain assessed by 11C-PMP PET in AD patients treated with galantamine. Neurobiol Aging. 2008;29: 168-184.

65. Dichgans M, Markus HS, Salloway S, Verkkoniemi A, Moline M, Wang Q, et al. Donepezil in patients with subcortical vascular cognitive impairment: A randomised double-blind trial in CADASIL. Lancet Neurol. 2008;7: 310-318.

66. Doody RS, Corey-Bloom J, Zhang R, Li H, Ieni J, Schindler R. Safety and tolerability of donepezil at doses up to 20 mg/day: Results from a pilot study in patients with Alzheimer's disease. Drugs Aging. 2008;25: 163-174.

67. Erkinjuntti T, Gauthier S, Bullock R, Kurz A, Hammond G, Schwalen S, et al. Galantamine treatment in Alzheimer's disease with cerebrovascular disease: Responder analyses from a randomized, controlled trial (GAL-INT-6). J Psychopharmacol. 2008;22: 761-768.

68. Jelic V, Haglund A, Kowalski J, Langworth S, Winblad B. Donepezil treatment of severe Alzheimer's disease in nursing home settings. A responder analysis. Dement Geriatr Cogn Disord. 2008;26: 458-466.

69. Kadir A, Darreh-Shori T, Almkvist O, Wall A, Grut M, Strandberg B, et al. PET imaging of the in vivo brain acetylcholinesterase activity and nicotine binding in galantamine-treated patients with AD. Neurobiol Aging. 2008;29: 1204-1217.

70. Moraes W, Poyares D, Sukys-Claudino L, Guilleminault C, Tufik S. Donepezil improves obstructive sleep apnea in Alzheimer disease: A double-blind, placebo-controlled study. Chest. 2008;133: 677-683.

71. Aronson S, Baelen BR, Kavanagh S, Schwalen S. Optimal dosing of galantamine in patients with mild or moderate Alzheimers disease: Post hoc analysis of a randomized, double-blind, placebo-controlled trial. Drugs and Aging. 2009;26: 231-239.

72. Ballard C, Brown R, Fossey J, Douglas S, Bradley P, Hancock J, et al. Brief psychosocial therapy for the treatment of agitation in Alzheimer disease (the CALM-AD trial). Am J Geriatr Psychiatry. 2009;17: 726-733.

73. Burns A, Bernabei R, Bullock R, Jentoft AJC, Frölich L, Hock C, et al. Safety and efficacy of galantamine (reminyl) in severe Alzheimer's disease (the SERAD study): A randomised, placebo-controlled, double-blind trial. Lancet Neurol. 2009;8: 39-47.

74. Grossberg G, Sadowsky C, Förstl H, Frölich L, Nagel J, Tekin S, et al. Safety and tolerability of the rivastigmine patch: Results of a 28-week open-label extension. Alzheimer Dis Assoc Disord. 2009;23: 158-164.

75. Cummings JL, Farlow MR, Meng X, Tekin S, Olin JT. Rivastigmine transdermal patch skin tolerability: Results of a 1-year clinical trial in patients with mild-to-moderate Alzheimers disease. Clinical Drug Investig. 2010;30: 41-49.

76. Farlow MR, Cummings JL, Olin JT, Xiangyi Meng. Effects of oral rivastigmine on cognitive domains in mild-to-moderate Alzheimer's disease. Am J Alzheimers Dis Other Demen. 2010;25: 347-352.

77. Farlow MR, Grossberg G, Gauthier S, Meng X, Olin JT. The ACTION study: Methodology of a trial to evaluate safety and efficacy of a higher dose rivastigmine transdermal patch in severe Alzheimer's disease. Curr Med Res Opin. 2010;26: 2441-2447.

78. Gauthier S, Lopez OL, Waldemar G, Jones RW, Cummings J, Zhang R, et al. Effects of donepezil on activities of daily living: Integrated analysis of patient data from studies in mild, moderate and severe Alzheimer's disease. Int Psychogeriatr. 2010;22: 973-983.

79. Jelic V, Darreh-Shori T. Donepezil: A review of pharmacological characteristics and role in the management of Alzheimer disease. Clinical Medicine Insights: Therapeutics. 2010;2: 771-788.

80. Seltzer B. Galantamine-ER for the treatment of mild-to-moderate Alzheimer's disease. Clin Interv Aging. 2010;5: 1-6.

81. Alva G, Grossberg GT, Schmitt FA, Meng X, Olin JT. Efficacy of rivastigmine transdermal patch on activities of daily living: Item responder analyses. Int J Geriatr Psychiatry. 2011;26: 356-363.

82. Burns A, Perry E, Holmes C, Francis P, Morris J, Howes MJ, et al. A double-blind placebo-controlled randomized trial of melissa officinalis oil and donepezil for the treatment of agitation in Alzheimer's disease. Dement Geriatr Cogn Disord. 2011;31: 158-164.

83. Farlow MR, Doraiswamy PM, Meng X, Cooke K, Somogyi M. The effect of vascular risk factors on the efficacy of rivastigmine patch and capsule treatment in Alzheimer's disease. Dement Geriatr Cogn Dis Extra. 2011;1: 150-162.

84. Gaudig M, Richarz U, Han J, van Baelen B, Schäuble B. Effects of galantamine in Alzheimer's disease: Double-blind withdrawal studies evaluating sustained versus interrupted treatment. Curr Alzheimer Res. 2011;8: 771-780.

85. Grossberg G, Meng X, Olin JT. Impact of rivastigmine patch and capsules on activities of daily living in Alzheimer's disease. Am J Alzheimers Dis Other Demen. 2011;26: 65-71.

86. Keller C, Kadir A, Forsberg A, Porras O, Nordberg A. Long-term effects of galantamine treatment on brain functional activities as measured by pet in Alzheimer's disease patients. J Alzheimer's Dis. 2011;24: 109-123.

87. Miettinen PS, Pihlajamäki M, Jauhiainen AM, Tarkka IM, Gröhn H, Niskanen E, et al. Effect of cholinergic stimulation in early Alzheimer's disease - functional imaging during a recognition memory task. Curr Alzheimer Res. 2011;8: 753-764.

88. Scarpini E, Bruno G, Zappalà G, Adami M, Richarz U, Gaudig M, et al. Cessation versus continuation of galantamine treatment after 12 months of therapy in patients with Alzheimer's disease: A randomized, double blind, placebo controlled withdrawal trial. J Alzheimer's Dis. 2011;26: 211-220.

89. Andersen F, Viitanen M, Halvorsen DS, Straume B, Wilsgaard T, Engstad TA. The effect of stimulation therapy and donepezil on cognitive function in Alzheimer's disease. A community based RCT with a two-by-two factorial design. BMC Neurol. 2012;12: doi: 10.1186/1471-2377-12-59.

90. Likitjaroen Y, Meindl T, Friese U, Wagner M, Buerger K, Hampel H, et al. Longitudinal changes of fractional anisotropy in Alzheimer's disease patients treated with galantamine: A 12-month randomized, placebo-controlled, double-blinded study. Eur Arch Psychiatry Clin Neurosci. 2012;262: 341-350.

91. McLaren DG, Sreenivasan A, Diamond EL, Mitchell MB, Dijk KR, Deluca AN, et al. Tracking cognitive change over 24 weeks with longitudinal functional magnetic resonance imaging in Alzheimer's disease. Neurodegener Dis. 2012;9: 176-186.

92. Farlow MR, Grossberg GT, Sadowsky CH, Meng X, Somogyi M. A 24-week, randomized, controlled trial of rivastigmine patch 13.3 mg/24 h versus 4.6 mg/24 h in severe Alzheimer's dementia. CNS Neurosci Ther. 2013;19: 745-752.

93. Ferris S, Karantzoulis S, Somogyi M, Meng X. Rivastigmine in moderately severe-to-severe Alzheimer's disease: Severe impairment battery factor analysis. Alzheimers Res Ther. 2013;5: 63.

94. Rountree SD, Atri A, Lopez OL, Doody RS. Effectiveness of antidementia drugs in delaying Alzheimer's disease progression. Alzheimer Dement. 2013;9: 338-345.

95. Sole-Padulles C, Bartres-Faz D, Llado A, Bosch B, Pena-Gomez C, Castellvi M, et al. Donepezil treatment stabilizes functional connectivity during resting state and brain activity during memory encoding in Alzheimer's disease. J Clin Psychopharmacol. 2013;33: 199-205.

96. Ohnishi T, Sakiyama Y, Okuri Y, Kimura Y, Sugiyama N, Saito T, et al. The prediction of response to galantamine treatment in patients with mild to moderate Alzheimer's disease. Curr Alzheimer Res. 2014;11: 110-118.

97. Pelton GH, Andrews H, Roose SP, Marcus SM, D'Antonio K, Husn H, et al. Donepezil treatment of older adults with cognitive impairment and depression (DOTCODE study): Clinical rationale and design. Contemp Clin Trials. 2014;37: 200-208.
